# Novel actions of arecoline in the *C. elegans* motor circuit

**DOI:** 10.17912/micropub.biology.000275

**Published:** 2020-07-01

**Authors:** Katherine A McCulloch, Yishi Jin

**Affiliations:** 1 Neurobiology Section, Division of Biological Sciences, University of California San Diego, La Jolla, CA 92093

**Figure 1:**
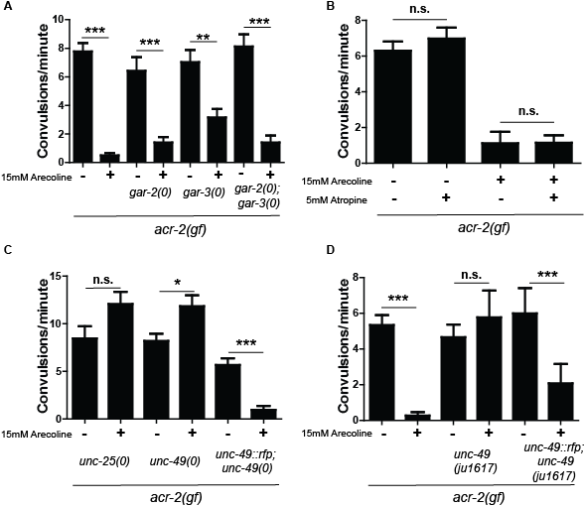
(A) The muscarinic receptors *gar-2* and *gar-3* are not required for suppression of *acr-2(gf)* by arecoline. (B) Pre-treatment with the specific muscarinic antagonist atropine does not affect suppression of *acr-2(gf*) by arecoline. (C) Loss of function in genes involved in GABAergic synaptic transmission eliminates suppression of *acr-2(gf)* by arecoline. (D) A new *unc-49(ju1617)* mutation was isolated in a genetic screen for mutants preventing suppression of *acr-2(gf)* by arecoline. Two-way ANOVA, ***P<0.001, **P<0.01, *P<0.05, n.s.=not significant.

## Description

*acr-2* encodes a nicotinic receptor subunit. A gain-of-function mutation in *acr-2* results in uncoordinated (Unc) movement and spontaneous shrinking or convulsion (Jospin, 2009). These phenotypes are the result of elevated cholinergic excitation as well as decreased GABAergic inhibition in the locomotor circuit. We have tested the effect of the muscarinic agonist arecoline on *acr-2(gf)* convulsion and found that this drug suppressed convulsion and locomotion phenotypes (McCulloch and Jin 2020). Here, we investigated underlying mechanisms and pathways involved in arecoline-induced suppression of *acr-2(gf)*.

There are two major types of muscarinic receptors. The M1/3/5 types are excitatory and act by elevating intracellular calcium. The M2/4 receptors are inhibitory and act by inhibiting cellular cAMP (Jones *et* *al.* 2012). *C. elegans* has three muscarinic receptors: *gar-1,* *gar-2* and *gar-3* (Hwang *et al.* 1999; Lee *et al.* 2000). Both *gar-2* and *gar-3* have been shown to respond to arecoline *in vivo.* *gar-3* is homologous to excitatory M1 muscarinic receptors, and promotes excitatory signaling in pharyngeal and motor circuits (Chan*et al.* 2013; Hwang *et al.* 1999; Kozlova *et al.* 2019; Liu *et al.* 2007; Steger and Avery 2004). *gar-2* is pharmacologically quite different from most muscarinic receptors, however, functionally, it seems to act similarly to inhibitory M2/4 type receptors in inhibiting cholinergic activity in the motor circuit (Dittman and Kaplan 2008; Lee *et al.* 2000). *gar-1*, unlike *gar-2* and *gar-3*, is expressed in the PVM neurons and other neurons in the head and has not been shown to be expressed in the motor circuit (Lee *et al.* 2000). We therefore focused on *gar-2* and *gar-3.* We constructed double and triple mutants of *acr-2(gf)* with null mutations in *gar-2* or *gar-3* (or mutations of both, respectively). Without arecoline, these mutants showed convulsion phenotypes indistinguishable from *acr-2(gf)* alone. Upon treatment with arecoline, we observed suppression of convulsion, similar to *acr-2(gf)* alone (Figure 1A). These genetic data suggested that these known muscarinic receptors in the motor circuit are not involved in suppression of *acr-2(gf)* by arecoline.

To further address if suppression of *acr-2(gf)* by arecoline involves muscarinic signaling, we tested atropine, which is a highly specific muscarinic antagonist and used to delineate muscarinic from non-muscarinic cholinergic effects (Rang, *et al.* 2012). It is used intravenously to treat patients with dangerously low heart rate, as inhibitory M2 receptors function in the heart to regulate cardiac activity. We treated *acr-2(gf)* animals with 5mM atropine for 2 hours, then transferred the animals to new plates with 5 mM atropine plus 15 mM arecoline for another 3 hrs and score them for convulsion. Atropine only, arecoline only, and untreated *acr-2(gf)* mutant animals were also scored as controls. However, atropine treatment did not alter the suppression of *acr-2(gf)* convulsion by arecoline (Figure 1B). This further supports a non-muscarinic mechanism for arecoline suppression of *acr-2(gf).*

Finally, reverse and forward genetic screens were employed to identify factors that are required for suppression of *acr-2(gf)* on arecoline. We tested multiple genes that are required for motor circuit function for their requirement for arecoline suppression. We found that null mutations in genes required for GABA signaling, such as *unc-25*/GAD and *unc-49*/GABAR, completely blocked arecoline suppression of *acr-2(gf)* (Figure 1C) (Bamber *et al.* 1999; Jin *et al.* 1999). Moreover, convulsion rates of these double mutants were slightly enhanced on arecoline. Null mutations in GABA genes strongly enhance the Unc phenotype of *acr-2(gf)* animals to paralysis, and this phenotype was also not suppressed on arecoline in the double mutants. These observations suggested that GABA signaling may be important for suppression of *acr-2(gf)* by arecoline.

In parallel to the reverse genetic screen, we conducted a forward genetic screen to identify factors involved in arecoline suppression of *acr-2(gf)* (see Reagents and Methods). We isolated three mutants that showed paralyzed phenotypes resembling GABA mutants, with or without arecoline treatment. Non-complementation analyses confirmed two of these to be *unc-49* mutations. Another mutation, *ju1617*, fully blocked arecoline suppression. However, *ju1617; acr-2(gf)* double mutants resembled *acr-2(gf)* in the absence of arecoline, in contrast to the *unc-49* null mutations. We isolated *ju1617* on its own, and these animals were slow-moving and exhibited loopy movement, somewhat similar to GABA mutants. After whole-genome sequencing analyses, we found a single nucleotide mutation altering a conserved Arg to His within a putative ligand-binding domain of UNC-49B (Bamber *et al.* 1999). To confirm the identity of *ju1617*, we constructed a *ju1617; acr-2(gf*) strain with an *unc-49* transgene *krSi2[Punc-49::unc-49::rfp]* and observed that these animals showed suppression of convulsion after arecoline treatment (Figure 1D). The *unc-49(ju1617)* mutation may represent a reduction-of-function mutation of *unc-49,* since it does not shrink in response to touch and fails to enhance *acr-2(gf)* Unc behaviors.

In summary, our studies from both forward and reverse genetic screens indicate that GABA signaling is required for suppression of *acr-2(gf)* by arecoline. One explanation is that arecoline is activating GABA signaling via an un-identified pathway to restore excitation and inhibition balance to the motor circuit, in the presence of *acr-2(gf).* Together, these data suggest that arecoline can act through novel means to inhibit cholinergic activity in specific contexts.

## Methods

Drug plates were prepared by supplementing standard NGM plates with drugs essentially as described for other *C. elegans* pharmacology assays (Mahoney *et al.* 2006). Drugs were dissolved in NGM at indicated concentrations prior to pouring. NGM-only plates were used as no-drug controls. All plates were seeded with a thin lawn of OP50 bacterial food. Two trials were performed for each experiment, with 10 animals in each trial (muscarinic agonists often drive animals to crawl off the plates, so some trials had <10 animals). Animals were transferred to drug plates and then scored after 3 hours. Convulsions were counted over 90s, and then normalized to 60s as convulsions per minute. For atropine studies, animals were incubated on NGM plates supplemented with 5mM atropine and scored for convulsion, or transferred to plates with 5mM atropine and 15mM arecoline, incubated for an additional 3hrs, and then scored for convulsion.

Genetic screening was performed using MT6241, essentially as described with modifications for drug-based isolation of mutants (Brenner 1974; McCulloch *et al.* 2017). F2s from mutagenized P0s were washed onto the center of 5mM arecoline plates and incubated for 3 hrs. Animals that had not moved from the center spot and visibly convulsed were picked, and then animals were analyzed in a second round, leaving 14 viable suppressors. Whole-genome sequencing analysis was performed as described (McCulloch *et al.* 2017).­­

## Reagents

**STRAINS**

**MT6241** *acr-2(n2420gf)* X

**CZ9381** *unc-25(n2328)* III; *acr-2(n2420gf)* X

**CZ9307** *unc-49(e382*) III; *acr-2(n2420gf)* X

**CZ9364** *gar-2(ok520)* III; *acr-2(n2420gf)* X

**CZ24303** *gar-3(gk305)* V; *acr-2(n2420gf)* X

**CZ25223** *gar-2(ok520)* III; *gar-3(gk305)* V; *acr-2(n2420gf)* X

**CZ26714** *unc-49(ju1617)* III

**CZ27187** *krSi2[Punc-49::unc-49::rfp]* II; *unc-49(e382)* III; *acr-2(n2420gf)* X

**CZ26795** *unc-49(ju1617)* III; *acr-2(n2420gf)* X

**CZ27978** *krSi2[Punc-49::unc-49::rfp]* II; *unc-49(ju1617)* III; *acr-2(n2420gf)* X

**Drugs used in this study:**

Arecoline hydrobromide, Acros Organics Cat#AC250130050

Atropine sulfate, Sigma Cat#A0257-256

## References

[R1] Bamber BA, Beg AA, Twyman RE, Jorgensen EM (1999). The Caenorhabditis elegans unc-49 locus encodes multiple subunits of a heteromultimeric GABA receptor.. J Neurosci.

[R2] Brenner S (1974). The genetics of Caenorhabditis elegans.. Genetics.

[R3] Chan JP, Staab TA, Wang H, Mazzasette C, Butte Z, Sieburth D (2013). Extrasynaptic muscarinic acetylcholine receptors on neuronal cell bodies regulate presynaptic function in Caenorhabditis elegans.. J Neurosci.

[R4] Dittman JS, Kaplan JM (2008). Behavioral impact of neurotransmitter-activated G-protein-coupled receptors: muscarinic and GABAB receptors regulate Caenorhabditis elegans locomotion.. J Neurosci.

[R5] Hwang JM, Chang DJ, Kim US, Lee YS, Park YS, Kaang BK, Cho NJ (1999). Cloning and functional characterization of a Caenorhabditis elegans muscarinic acetylcholine receptor.. Receptors Channels.

[R6] Jin Y, Jorgensen E, Hartwieg E, Horvitz HR (1999). The Caenorhabditis elegans gene unc-25 encodes glutamic acid decarboxylase and is required for synaptic transmission but not synaptic development.. J Neurosci.

[R7] Jones CK, Byun N, Bubser M (2011). Muscarinic and nicotinic acetylcholine receptor agonists and allosteric modulators for the treatment of schizophrenia.. Neuropsychopharmacology.

[R8] Jospin M, Qi YB, Stawicki TM, Boulin T, Schuske KR, Horvitz HR, Bessereau JL, Jorgensen EM, Jin Y (2009). A neuronal acetylcholine receptor regulates the balance of muscle excitation and inhibition in Caenorhabditis elegans.. PLoS Biol.

[R9] Kozlova AA, Lotfi M, Okkema PG (2019). Cross Talk with the GAR-3 Receptor Contributes to Feeding Defects in *Caenorhabditis elegans eat-2* Mutants.. Genetics.

[R10] Lee YS, Park YS, Nam S, Suh SJ, Lee J, Kaang BK, Cho NJ (2000). Characterization of GAR-2, a novel G protein-linked acetylcholine receptor from Caenorhabditis elegans.. J Neurochem.

[R11] Liu Y, LeBoeuf B, Garcia LR (2007). G alpha(q)-coupled muscarinic acetylcholine receptors enhance nicotinic acetylcholine receptor signaling in Caenorhabditis elegans mating behavior.. J Neurosci.

[R12] Mahoney TR, Luo S, Nonet ML (2006). Analysis of synaptic transmission in Caenorhabditis elegans using an aldicarb-sensitivity assay.. Nat Protoc.

[R13] McCulloch Katherine A, Jin Yishi (2020). The muscarinic agonist arecoline suppresses motor circuit hyperactivity in *C. elegans*. microPublication Biology.

[R14] Steger KA, Avery L (2004). The GAR-3 muscarinic receptor cooperates with calcium signals to regulate muscle contraction in the Caenorhabditis elegans pharynx.. Genetics.

[R15] Rang, H. P., M. M. Dale, J. M. Ritter, and R. J. Flower. 2012. <i>Rang and Dales pharmacology</i>. Philadelphia: Elsevier Churchill Livingstone.

